# Predictors of immune reconstitution inflammatory syndrome associated with Kaposi’s sarcoma: a case report

**DOI:** 10.1186/s13027-016-0051-3

**Published:** 2016-02-03

**Authors:** Anna Maria Cattelan, Adriana Mattiolo, Angela Grassi, Maria Assunta Piano, Lolita Sasset, Marco Trevenzoli, Paola Zanovello, Maria Luisa Calabrò

**Affiliations:** Infectious and Tropical Diseases, Azienda Ospedaliera and University of Padova, Padova, Italy; Immunology and Molecular Oncology, Veneto Institute of Oncology, IOV IRCCS, Padova, Italy; Infectious Diseases, ULSS 18 - Azienda Ospedaliera, Rovigo, Italy; Department of Infectious and Tropical Diseases, University of Padova, School of Medicine, Padova, Italy; Department of Surgery, Oncology and Gastroenterology, University of Padova, Padova, Italy

**Keywords:** Immune reconstitution inflammatory syndrome, IRIS, Kaposi’s sarcoma, KS, KS-IRIS, HHV8, KSHV, Biomarker

## Abstract

We present here a case of immune reconstitution inflammatory syndrome associated with Kaposi’s sarcoma (KS-IRIS) developed in an AIDS patient two months after initiation of antiretroviral therapy (ART). Baseline characteristics of this IRIS-KS case, within a cohort of 12 naïve AIDS-KS patients, were analyzed. No statistically significant differences in CD4 cell counts, plasma HIV RNA load, KS clinical staging, human herpesvirus 8 (HHV8) antibody titers and HHV8 load in peripheral blood mononuclear cells and saliva were evidenced. HHV8 load in plasma was found to be significantly higher in the KS-IRIS patient (> 6 log_10_ genome equivalents/ml, *p* = 0.01, *t–*test) compared to the 11 patients with KS regression. This case highlights that measurement of HHV8 load in plasma may be useful to identify patients at risk for KS-IRIS, and that this parameter should be included in the design of larger studies to define KS-IRIS risk predictors.

## Introduction

Although it was initially reported in HIV-negative patients, the immune reconstitution inflammatory syndrome (IRIS) occurs more frequently in HIV-infected patients initiating antiretroviral therapy (ART). IRIS results from restored immunity causing an exaggerated inflammatory response against persistent infectious or non-infectious antigens [1–5] and occurs with a wide spectrum of clinical manifestations. IRIS may cause worsening of previously treated infections, tumor progression in patients with proliferative disorders, or unmasking of subclinical infections, with tumor development in patients co-infected with an oncogenic virus. One of the parameters predictive of IRIS is severe CD4 cell lymphopenia prior to ART initiation (< 50 cells/μl). Other relevant biomarkers are the elevated antigenic burden of an opportunistic infection and a decrease of at least more than 1 log_10_ HIV RNA in plasma, which represents one of the major criteria for the definition of IRIS [1, 2, 5].

A subset of patients (6–20 %) co-infected with human herpesvirus 8 (HHV8) may develop Kaposi’s sarcoma (“unmasking” Kaposi’s sarcoma-associated IRIS, KS-IRIS) or experience acute worsening of pre-existing tumor (“paradoxical” KS-IRIS) within a short interval (2–3 months) after ART initiation. The clinical course of KS-IRIS varies greatly according to the analyzed population, as demonstrated by Letang et al. [6] in a pooled analysis of 436 AIDS-KS patients initiating ART from two distinct geographical areas, UK and sub-Saharan Africa. KS-IRIS can be self-limiting in well-resourced countries, with a tendency to regress even without therapy readjustment. On the other hand, this condition is frequently associated with high mortality in sub-Saharan countries, reflecting the more advanced stage at presentation and the higher frequencies of comorbidities and coinfections in African populations. The study by Letang et al. [6] identified the following baseline biomarkers for risk stratification: ART alone as initial KS treatment, T_1_ KS clinical stage, and high plasma HIV RNA load (> 5 log_10_ copies/ml). Moreover, detectable baseline plasma HHV8 DNA was also found to predict KS-IRIS development in a subset of 259 patients, whereas quantification of plasma viral load showed no significant differences. However, the IRIS condition is characterized by an immunological disturbance in untreated AIDS-KS patients, leading to systemic activation of HHV8 lytic replication with subsequent viremia. Thus, baseline systemic viral burden might be useful for risk stratification, as previously demonstrated in IRIS associated with opportunistic infections and in AIDS-KS patients, in which a better clinical response was found to be correlated to a baseline plasma HHV8 load below 660 copies/ml [7].

## Case report

A 39-year-old HIV-positive homosexual male was admitted to our Infectious Diseases Unit with asthenia and multiple (11) painless, nodular purple lesions concentrated in the trunk that ranged from 0.5 to 2.0 cm in the largest dimension. The patient was diagnosed with HIV infection and cutaneous Kaposi’s sarcoma was confirmed by histological examination. Either mucocutaneous and visceral involvement was excluded by endoscopic and neck, chest and abdominal computerized tomography (CT) examinations. The CD4 lymphocyte count was 360 cells/μl (14 %) and plasma HIV-1 RNA level was 4.09 log_10_ copies/ml. Parameters linked to HHV8 infection status, evaluated as previously reported [8–10], revealed active lytic replication, with high burden of cell-free viral progeny in plasma [6.16 log_10_ genome equivalents (GE)/ml] and saliva (4.95 log_10_ GE/ml, Table 1). The patient started a combined ART (cART) with darunavir/ritonavir plus coformulated tenofovir/emtricitabine. Four weeks after cART initiation, examination of the patient revealed stable cutaneous KS lesions; the CD4 lymphocyte count had increased to 810 cells/μl (20 %) and the plasma HIV RNA was undetectable (< log_10_ 40 copies/ml). Within a further 4 week-treatment, the patient showed recrudescent KS, with an increased number of lesions (> 20 cutaneous lesions) and enlargement of pre-existing ones. CD4 cell count was 820 cells/ml (21 %) and HIV RNA viral load was under the limit of detectability. The sudden clinical KS worsening together with the rapid immunovirological response was indicative of an immune reconstitution inflammatory syndrome rather than KS progression. The patient continued the antiretroviral combination therapy and no systemic KS chemotherapy was administrated. KS-IRIS flare completely recovered in 10 months.

**Table 1:** Characteristics of the 12 male KS patients at study entry

	KS-IRIS (*N* = 1)	KS (*N* = 11)^a^
Demographics		Median (IQR)
Age (years)	39	35 (31.5–53)
Risk factor		N
Homosexual transmission	1	9
Bisexual transmission		1
Heterosexual transmission		1
KS staging		
T_0_I_0_S_0_	1	1
T_1_I_0_S_0_		3
T_1_I_0_S_1_		2
T_0_I_1_S_1_		1
T_1_I_1_S_1_		4
HIV- and HHV8-related parameters		Median (IQR)
CD4 cell count (cells/μl)	360	190 (80–335)
HIV plasma load (log_10_ copies/ml)	4.09	4.97 (3.89–5.31)
HHV8 PBMC load (GE/10^5^ cells)	76	100 (26.50–160.75)
HHV8 plasma load (log_10_ GE/ml)	6.16^b^	3.48 (3.26–3.76)
HHV8 saliva load (log_10_ GE/10^5^ cells)	4.95	2.87 (1.84–4.14)
HHV8 ORF65 antibodies^c^	< 50	400 (100–800)
HHV8 LANA antibodies^c^	12,800	800 (250–4,800)

*KS-IRIS* Kaposi’s sarcoma-associated immune reconstitution inflammatory syndrome, *KS* Kaposi’s sarcoma, *IQR* interquartile range, *HHV8* human herpesvirus 8, *PBMC* peripheral blood mononuclear cells, *GE* genome equivalents, *ORF65* open reading frame 65, encoding for a structural protein expressed during the lytic phase, *LANA* latency-associated nuclear antigen. ^a^In the KS group, data are expressed as median (interquartile range) calculated on the measurable samples. ^b^Significantly higher in the KS-IRIS case *vs.* KS group (*p* = 0.01). To perform statistical analysis, samples in which HIV RNA (*N* = 2) could not be detected were assigned an arbitrary value of log_10_40, specimens in which HHV8 ORF65 antibodies (*N* = 3) were not detected an arbitrary value of 40, and those in which HHV8 GE were not detected (PBMC, *N* = 3; plasma, *N* = 1; saliva, *N* = 1) were assigned an arbitrary value of 5 (PBMC) or log_10_5. ^c^Antibody titers were determined using 1:50 as first dilution, and are expressed as reciprocal of the highest dilution giving a positive result

To test the usefulness of previously reported KS-IRIS predictors [6], we analyzed baseline characteristics of the preceding 11 consecutive cART-naïve AIDS-KS patients referring to the Infectious Diseases Units of Padova and Rovigo, and followed at the Veneto Institute of Oncology for HHV8 diagnostics. All 11 patients had histologically confirmed KS. They had initiated combined antiretroviral therapy (cART) showing immunovirological response, accompanied by KS regression (data not shown), during the first 3 months of follow-up. Baseline characteristics of all KS patients are reported in Table 1. In the KS group, data are expressed as median and interquartile range (25^th^–75^th^ percentile) calculated on the measurable samples. To compare the values of different biomarkers of the KS-IRIS case to those of the KS group, the one-tailed Crawford and Howell *t*-test was used [11]. No statistically significant differences in CD4 cell counts, plasma HIV RNA load, KS clinical staging [12], HHV8 antibody titers and HHV8 load in PBMC were observed. Conversely, HHV8 load in plasma was measurable in 11 of 12 patients, and was found to be significantly higher in the KS-IRIS case (> 6 log_10_ GE/ml, *p* = 0.01) compared to those measured in patients with KS regression. Moreover, HHV8 load in saliva was higher, albeit not statistically significant (*p* = 0.09), in the KS-IRIS case (Table 1).

## Discussion

These findings, although limited to a small cohort, indicate that plasma HHV8 DNA detectability may not be so informative, and are fully in line with previously published studies. Indeed, 78.4 % of ART-naïve AIDS-KS patients from Zimbabwe had measurable plasma load, with a baseline median value of 2.82 log_10_ copies/ml [6, 7]. Moreover, HHV8 viremia was detectable at study entry in more than 90 % of AIDS-KS patients belonging to the Chelsea and Westminster HIV cohort, one of the largest cohorts in Europe, with a baseline median HHV8 load ranging from 4.6 to 5 log_10_ copies/ml [13]. The same cohort was analyzed in the pooled analysis [6], and plasma HHV8 DNA detectability, determined only in 104 patients, dropped to 58.3 % in this selected population, as did the baseline median value (2.87 log_10_ copies/ml) of plasma HHV8 load. We think that these discrepancies might be due to different sensitivities in the quantitative techniques, if samples were independently reanalyzed, or to a unrecognized bias related to the selection of the 104 patients for the pooled analysis. Another population of AIDS-KS patients included in Letang et al. [6] is the Durban cohort, from South Africa [14]; about half of these samples (54/112) were analyzed in the pooled analysis, and 74.1 % of the samples have measurable plasma HHV8 load, with a baseline median value of 2.24 log_10_ copies/ml [6]. Therefore, most of the individual studies showed high frequencies of HHV8 detectability in plasma, as did our small cohort; these figures are far from the highest, measured or estimated, incidences of KS-IRIS in any analyzed population, indicating that the HHV8 detection *per se* cannot be a predictive parameter.

## Conclusion

This case highlights that, in our small cohort, the measurement of baseline HHV8 load in plasma was useful to identify the patient who experienced a KS-IRIS. Therefore, we recommend including quantification of baseline parameters linked to HHV8 infection status, specifically measurement of HHV8 load in plasma and, possibly, in saliva, in the design of larger studies to define KS-IRIS predictors.

## Consent

This study was evaluated and approved by the Ethical Committee of the Veneto Institute of Oncology (Protocol n. 14256), and written informed consent was obtained from study participants.

## Abbreviations

ART: antiretroviral therapy
cART: combined ART
GE: genome equivalents
HAART: highly active antiretroviral therapy
HHV8: human herpesvirus 8
IQR: interquartile range
IRIS: immune reconstitution inflammatory syndrome
KS: Kaposi’s sarcoma
KS-IRIS: immune reconstitution inflammatory syndrome associated with KS
LANA: latency-associated nuclear antigen
ORF65: open reading frame 65
PBMC: peripheral blood mononuclear cells
